# INSTRUCTIONAL DESIGN FOR INTERGENERATIONAL AND AGE-INCLUSIVE LEARNING: LESSONS FROM A GERONTOLOGY CLASSROOM

**DOI:** 10.1093/geroni/igad104.0083

**Published:** 2023-12-21

## Abstract

The paper will present a multi-year research study that examined the learning needs of first-generation younger and older learners in a graduate-level online learning environment. Ageism and related stereotyping will be reviewed as concepts that reflect a potentially biased understanding of all age groups that should be addressed in intergenerational learning activities. Students’ perceptions of understanding aging issues and stereotypes were compared pre- and post-course. A model will be presented about designing an age-inclusive, age-friendly online course design utilizing intergenerational learning activities to optimize learning outcomes while reducing ageist assumptions through targeted virtual discussions about diversity perceptions in the classroom—the design of intergenerational engagement discussion and other learning assignment activities in an introductory graduate gerontology course. Implications for creating intergenerational programming will be discussed.

